# Biologic Rhythms Derived from Siberian Mammoths' Hairs

**DOI:** 10.1371/journal.pone.0021705

**Published:** 2011-06-29

**Authors:** Mike Spilde, Antonio Lanzirotti, Clifford Qualls, Genevieve Phillips, Abdul-Mehdi Ali, Larry Agenbroad, Otto Appenzeller

**Affiliations:** 1 Department of Earth and Planetary Sciences, Institute of Meteoritics, University of New Mexico, Albuquerque, New Mexico, United States of America; 2 National Synchrotron Light Source, Brookhaven National Laboratory, Upton, New York, United States of America; 3 Departments of Mathematics and Statistics, University of New Mexico, Albuquerque, New Mexico, United States of America; 4 Fluorescence Microscopy Facility, Cancer Research and Treatment Center, University of New Mexico, Albuquerque, New Mexico, United States of America; 5 Department of Earth and Planetary Sciences, Analytical Chemistry Laboratory, University of New Mexico, Albuquerque, New Mexico, United States of America; 6 The Mammoth Site, Hot Springs, South Dakota, United States of America; 7 New Mexico Health Enhancement and Marathon Clinics Research Foundation, Albuquerque, New Mexico, United States of America; University of Maribor, Slovenia

## Abstract

Hair is preserved for millennia in permafrost; it enshrines a record of biologic rhythms and offers a glimpse at chronobiology as it was in extinct animals. Here we compare biologic rhythms gleaned from mammoth's hairs with those of modern human hair. Four mammoths' hairs came from varying locations in Siberia 4600 km, four time zones, apart ranging in age between 18,000 and 20,000 years before present. We used two contemporaneous human hairs for comparison. Power spectra derived from hydrogen isotope ratios along the length of the hairs gave insight into biologic rhythms, which were different in the mammoths depending on location and differed from humans. Hair growth for mammoths was ∼31 cms/year and ∼16 cms/year for humans. Recurrent annual rhythms of slow and fast growth varying from 3.4 weeks/cycles to 8.7 weeks/cycles for slow periods and 1.2 weeks/cycles to 2.2 weeks/cycles for fast periods were identified in mammoth's hairs. The mineral content of mammoth's hairs was measured by electron microprobe analysis (k-ratios), which showed no differences in sulfur amongst the mammoth hairs but significantly more iron then in human hair. The fractal nature of the data derived from the hairs became evident in Mandelbrot sets derived from hydrogen isotope ratios, mineral content and geographic location. Confocal microscopy and scanning electron microscopy showed varied degrees of preservation of the cuticle largely independent of age but not location of the specimens. X-ray fluorescence microprobe and fluorescence computed micro-tomography analyses allowed evaluation of metal distribution and visualization of hollow tubes in the mammoth's hairs. Seasonal variations in iron and copper content combined with spectral analyses gave insights into variation in food intake of the animals. Biologic rhythms gleaned from power spectral plots obtained by modern methods revealed life style and behavior of extinct mega-fauna.

## Introduction

Hair is often well preserved for centuries; it resists decay especially in Siberian permafrost where it can be conserved for millennia.

Modern sequencing has spawned remarkable advances in the study of ancient DNA. These include the sequencing of 28 million base pairs of mammoth DNA, which established that this behemoth split from its African elephant cousin about 6 million years ago. The physiological underpinnings of life in the cold Siberian winter, where mammoths roamed, have also been revealed by the special hemoglobin sequence found in mammoth DNA and subsequently reproduced in modern bacteria [1]. These studies showed that substitutions in hemoglobin confer biochemical properties adaptive for cold-tolerance. The genetic material from which these details were gleaned originated in mammoth hair found in permafrost [2].

In multicellular organisms, with functioning nervous systems, clock-like signals originate in the anterior part of the hypothalamus; the “master time keeper” of the brain; these signals drive biologic rhythms. These rhythms, in turn, are paced by changes in gene expression, which then drive intracellular clock-proteins which are found in all tissues. The signals reach the peripheral tissues, such as hair, through the autonomic nervous system (ANS), a part of the nervous system that is independent of volitional control and, ultimately, this affects hair growth [3]. To understand the feeding behavior and survival strategies of extinct vertebrates such as mammoths, it is necessary to understand their energy requirements and their capacities to cope with varying climates [4]. The daily light-dark cycle affects numerous aspects of physiology through the circadian clock and the Siberian climate is greatly affected by its high latitude and months-long uninterrupted darkness. Many organs, including hair, have their own clocks that function independently of the master clock in the brain's hypothalamus [5]. The human circadian clock has been studied in hair follicles, the bulbous attachment at the root of the hair [6]. The circadian cycles of clock genes are accurately reflected in the behavioral rhythms of the subjects. Even in shift workers, who have deranged expressions of clock genes, which are out of phase with their life styles, the oscillations of the genes in their hair roots mirror their unusual life style.

Using spectral analysis of the variation in hydrogen isotope ratios along the length of the hair allows assessment of biologic rhythms in animals and gives insight into their physiology. These vary depending on geography, as different environments contain slightly different ratios. As a result the hydrogen isotope ratios preserved in hair or feathers can trace an animal's migrations over its lifetime [3].

We thought to explore the effects on biorhythms of the prolonged darkness that prevails in Siberia and the wide geographic separation of the terrain where mammoths roamed on the chronobiology of these animals. We also used the same techniques on human hair for comparisons of the effects of different body size and usual circadian light-dark cycles on hair-derived chronobiology.

Here we report on biologic rhythms of wooly mammoths using the variation in hydrogen isotope composition of their hairs and from their elemental content.

## Results

**Figure 1:**
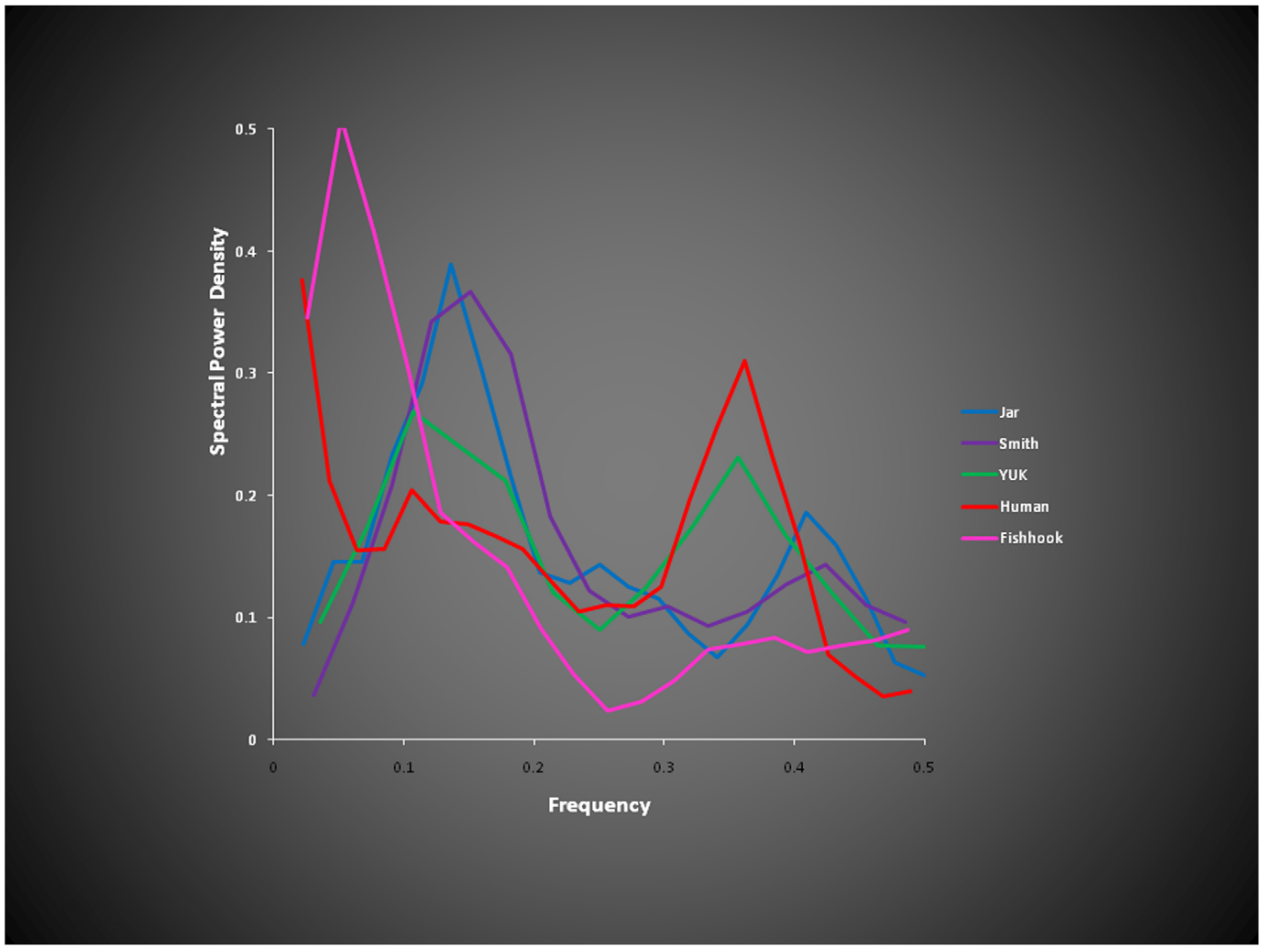
Power spectra derived from hydrogen isotope ratios along the hair from a human (red) and from mammoths identified by colors. The spectra contain low and high frequencies but their power varies across species and mammoth locations. The spectra are similar in shape, but not power, to those derived from human heart rate variability.

Wooly mammoth hairs came from Northern Siberia. They were labeled Jar, Smith, YUK and Fish. Jar and YUK were found ∼4600 km apart spanning four time zones. The hairs varied in age carbon dated to between 18,000 and 20,000 years ago. Human hair from contemporaneous individuals, one young woman from Italy and the other from an elderly US male were also analyzed. Differences were seen in the hydrogen-isotope-derived power spectra of the hairs reflecting the different biologic rhythms of the animals and humans (Fig. 1).

A comparison of the power spectra derived from the hydrogen isotope ratios of the hairs (Fig. 1) shows that the low and high frequencies of the spectra differ considerably in power amongst the 4 mammoths and are also markedly different from the spectrum obtained from the woman's hair.

Mammoth hair grows approximately 31 cms./year [7]. By contrast human hair grows only, on average, 16 cms./year. These differences in growth rate are most likely due to the thermoregulatory demands and life styles of mammoth' in Northern Siberia [3].

**Figure 2:**
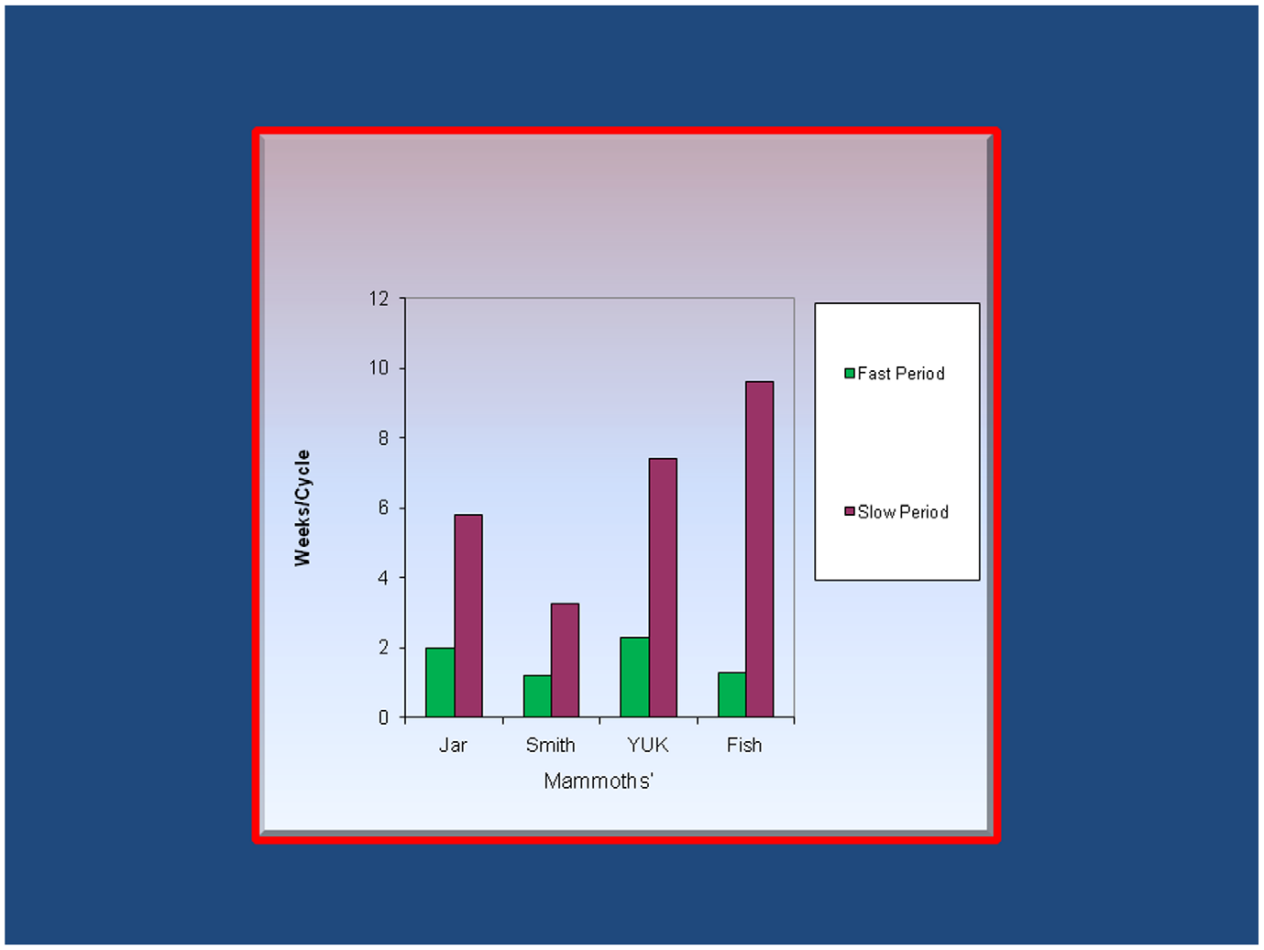
Fast and slow annual periods of hydrogen isotope ratios from mammoth hair. The fast periods are markedly faster than in human hair consistent with faster growth rate and appropriate thermoregulation in the Siberian climate. The slow period cycling differs amongst the 4 locations and is also remarkably accelerated in the mammoth. Note that the slow periods of Smith (3.25 weeks) and YUK (7.4 weeks) may reflect the geographic separation of these animals (spanning over ∼4600 Km in longitude) and the different isoscapes along this great distance.

The spectral indices of the hydrogen isotope ratios along the length of the mammoth's hairs were used to identify recurring annual rhythms of slow and fast periods (Fig. 2). The slow periods varied from 3.4 weeks/cycles (Smith) to 8.7 weeks/cycles (FISH); the fast periods from 1.2 weeks/cycles (Smith and FISH) to 2.2 weeks/cycles (YUK).

**Figure 3:**
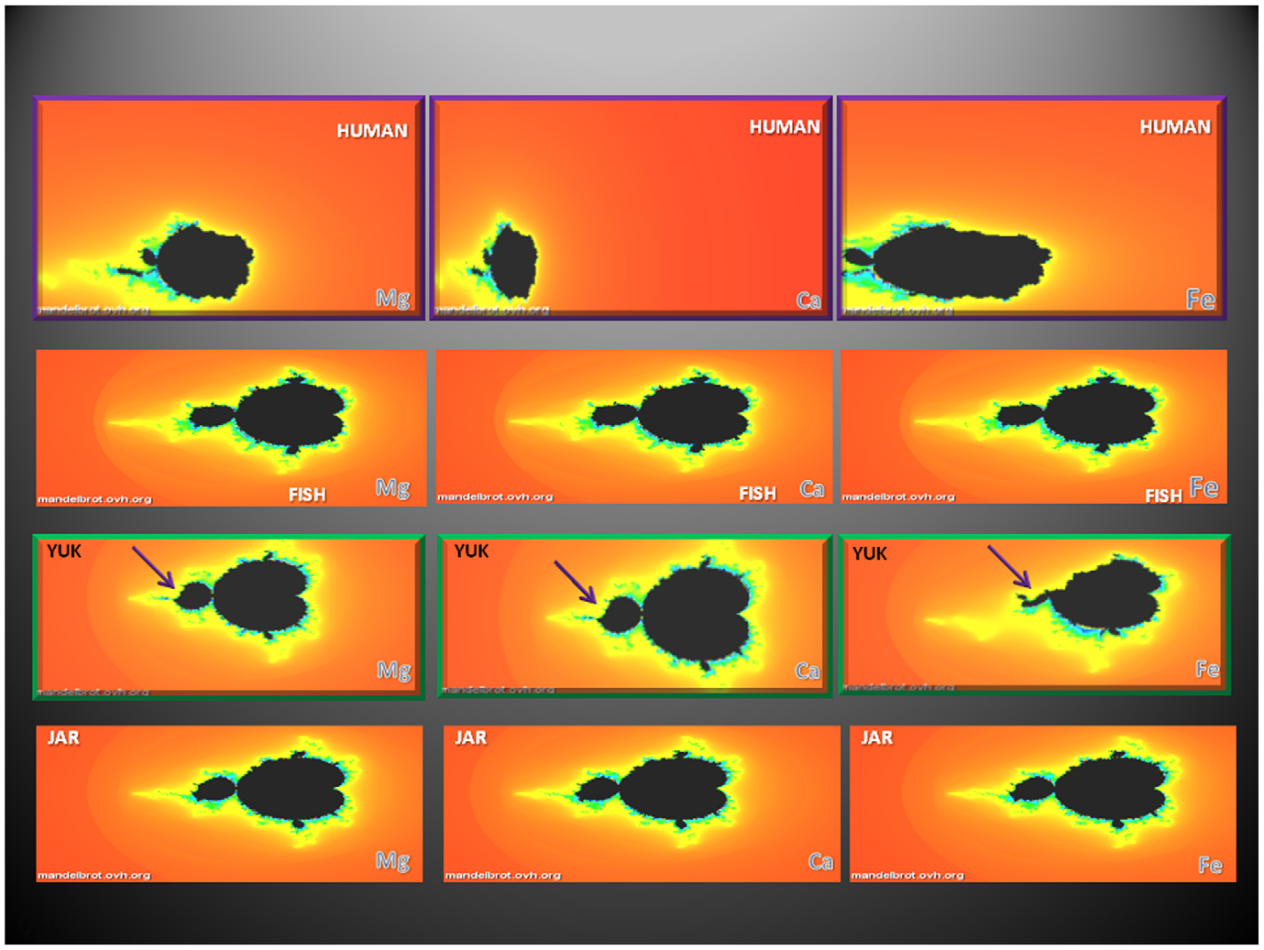
Mandelbrot sets derived from one human and three mammoth's hairs found at geographically separate locations in Siberia. All sets were derived using the data for power spectra obtained from the hydrogen isotope ratios along the length of the hairs and the hair content of sulfur and other elements (Mg, Ca, and Fe), using Mandelbrot set-on line generator (© 2007–2011 Dawid Makieła) (Mandelbrot.ovh.org). The sets depicting the fractals for each hair, using different elements, were similar in contrast to the set generated for the YUK hair, which shows marked deviations from the usual forms seen in FISH and JAR (arrows). The human fractals are noticeably different although the same parameters as those for the mammoths were used. All images were 30% brightness and 10% contrast enhanced.

To determine the relationships between the power spectra derived from hydrogen isotope ratios (proxies for biologic rhythms), the mineral content of the mammoths' hairs (a reflection of diet) and the different geographical locations where the specimens were found we turned to Mandelbrot fractal analysis. The data are infinitely complex and thus lend themselves to such analysis (Fig. 3). The most remarkable differences in the Mandelbrot sets were found in the YUK mammoth. Notably, the fractal sets for Mg, Ca, and Fe were not different for each hair, individually, implying that the power spectra reflecting biologic rhythms and the geographic locations and not the intake of elements were the predominant drivers of the Mandelbrot fractal sets (Fig. 3).

**Figure 4:**
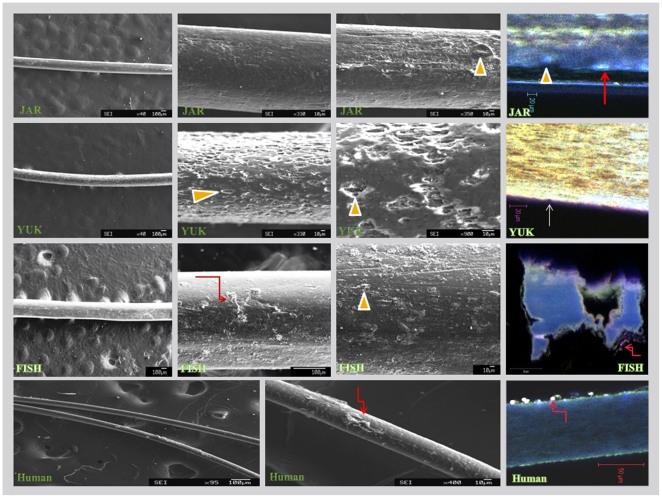
Confocal and scanning microscopic images of mammoth's and human hair. Taphonomic changes, indicated by arrow-heads and straight white arrow in the confocal image. Bacterial-fungal colonies are shown by broken arrows. Red arrow indicates space between cuticle and hair shaft due to shrinkage. Extensive taphonomic changes in YUK are evident. Note the preservation of the cuticle in the human hair in contrast to the craters in the cuticles of the mammoth's hair. Bacterial biofilms are a feature of human hair; mammoth's hair was protected from bacterial invasion in permafrost.

We next examined the hairs using confocal microscopy (Fig. 4). The preservation of the hairs varied greatly with location, while age was a lesser factor. The YAR and FISH hairs were of approximately the same age yet showed different stages of deterioration of the cuticle, the outer tough shell of the hair, as evidenced by the destruction of this structure from large areas of FISH's hair and the relative preservation of the cuticle in JAR. The human hair was characterized by preservation of the cuticle but marked invasion by bacteria shown in extensive biofilms, largely absent from the mammoths' hairs. This implies that permafrost was, as expected, protective from bacterial invasion and contributed to the millennia-long preservation of the mammoth's hairs.

To appraise further taphonomic effects (the conditions and processes that affect tissues before they become stabilized for millennia) on the mammoths' hairs we used scanning electron microscopy (SEM) (Fig. 4). This revealed large craters in YUK and surprisingly less “cratering” in YAR and FISH, the older hairs (Fig. 4). The confocal images confirmed the SEM results (Fig. 4).

**Figure 5:**
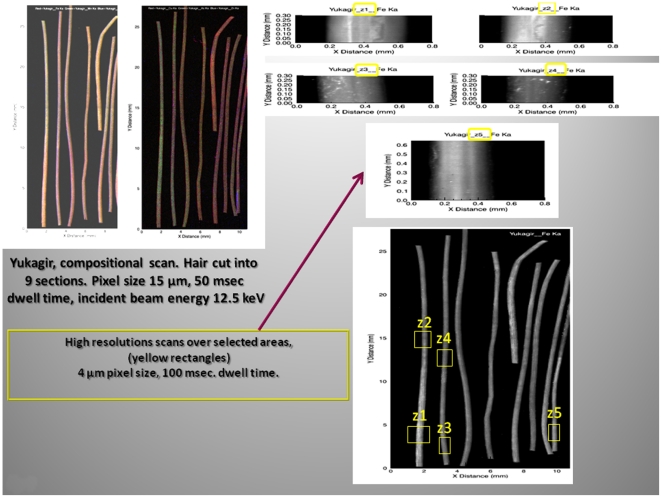
Micro-focused synchrotron X-ray fluorescence images. Compositional scan and high resolution scans of selected areas of the Yuk mammoth hair. Note the focal accumulation (z1–z5) of Fe along the length of the hair.

**Figure 6:**
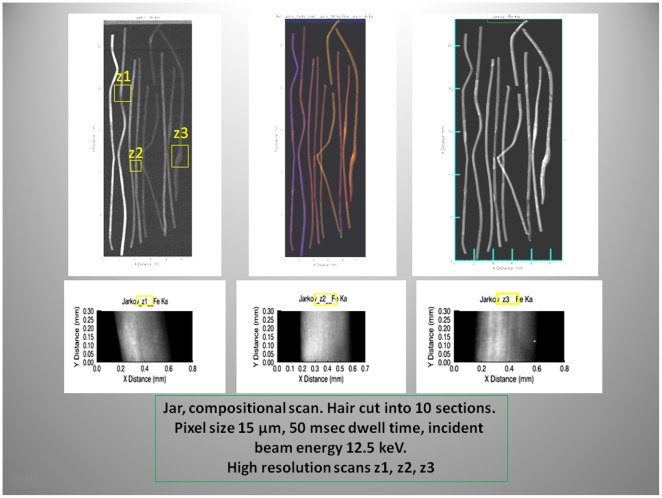
Micro-focused synchrotron X-ray fluorescence images. Compositional scan and high resolution scans of selected areas of the Jar mammoth hair. Note the focal accumulation of Fe (z1, z2, z3) along the length of the hair; high resolution scans, lower panels (yellow rectangles).

**Figure 7:**
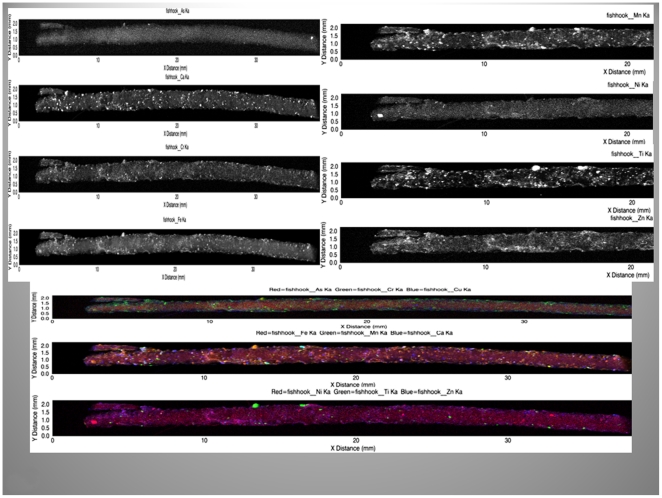
Micro-focused synchrotron X-ray fluorescence images. Overview of entire Fish hair, showing the focal accumulation of various elements. Note the distribution along the cuticle of Ti and the diffuse occurrence of Fe.

**Figure 8:**
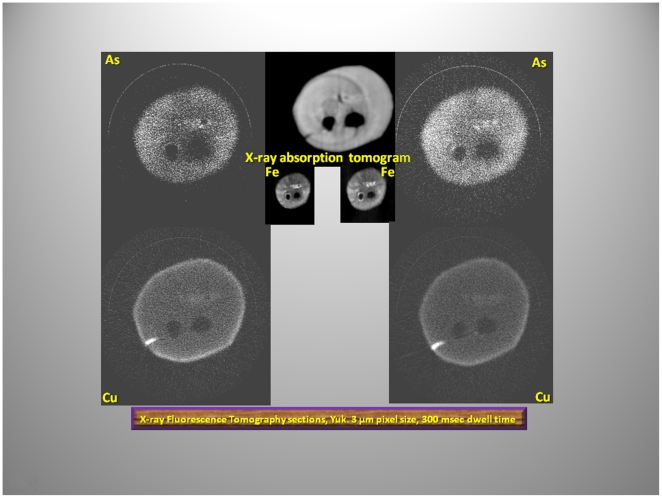
X-ray fluorescence tomography of selected elements in the YUK hair. Note: a) the double air spaces in the hair, a feature of the thermoregulatory function of the hair. b) in the grayscale images, whiter areas reflect higher elemental concentrations. c) the focal distribution of Fe and Cu contrasts to the diffuse dispersal of As throughout this hair.

To investigate the elemental composition we examined the mammoths' hairs using the X-ray fluorescence microprobe at the National Synchrotron Light Source (beam line X26A), Brookhaven National Laboratory Upton, NY, USA. Two-dimensional fluorescence compositional maps for selected elements such as Fe, As, and Mn were generated to construct plots of the fluorescence intensity versus the lengths of the hairs for the YUK and JAR (Figs. 5, 6). Additionally, an overview for the Fish hair is given in Fig. 7. Small sections of the hairs were analyzed by X-ray fluorescence computed micro-tomography to show the three-dimensional distribution of trace metals in the reconstructed cross-sections through the hair (Fig. 8). This allowed qualitative evaluation of metal distribution within the hair. Arsenic is uniformly distributed; Cu is increased at the hair's outer layers and then uniformly distributed internally while Fe shows localized variability in abundance within the hair. Reconstruction of X-ray attenuation through the samples also shows the presence of void spaces within the hairs; the air-tubes (Fig. 8).

**Figure 9:**
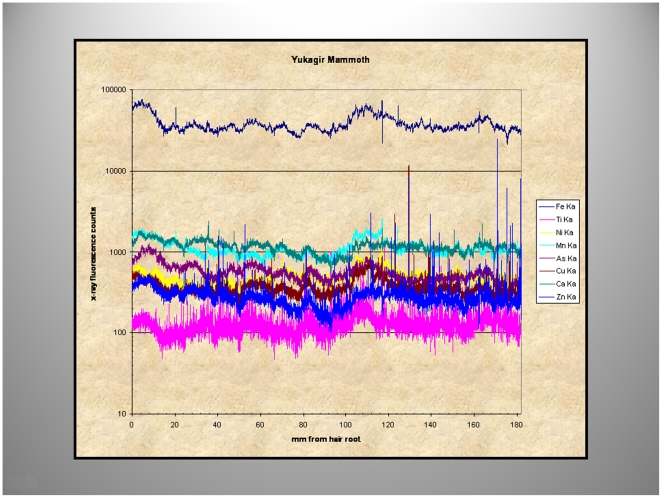
Micro-focused synchrotron X-ray fluorescence analyses of Yuk[agir] mammoth hair. Note the large preponderance of Fe compared to the other elements (logarithmic scale).

Plots for fluorescence intensity for Fe, Ti, Ni, Mn, As, Cu, Ca, and Zn and the distance from the hair root for Yuk's hair are shown in Fig. 9. This plot demonstrates the abundance of Fe over the other elements on a logarithmic scale and the periodic synchronous increases in all elemental content in the YUK hair plotted along the length of the hair (Fig. 9).

**Figure 10:**
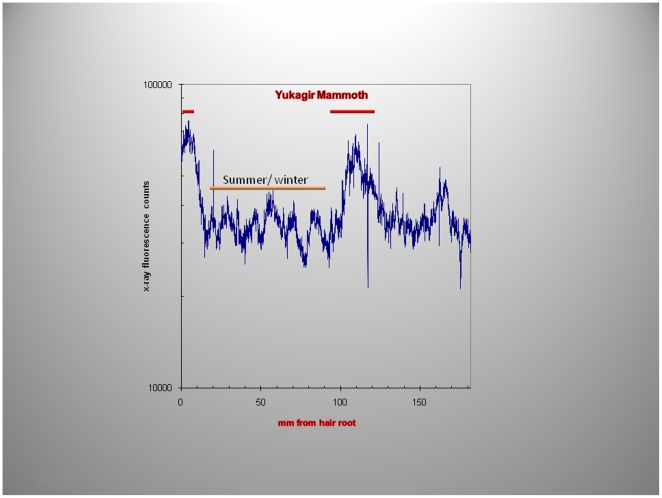
Micro-focused synchrotron X-ray fluorescence analyses of Yuk[agir] mammoth hair. During the summer/winter months (orange line), the amount of Fe is lower than that which occurs during spring and autumn (red lines); a reflection of the increased food intake during these relatively short periods in Northern Siberia.

**Figure 11:**
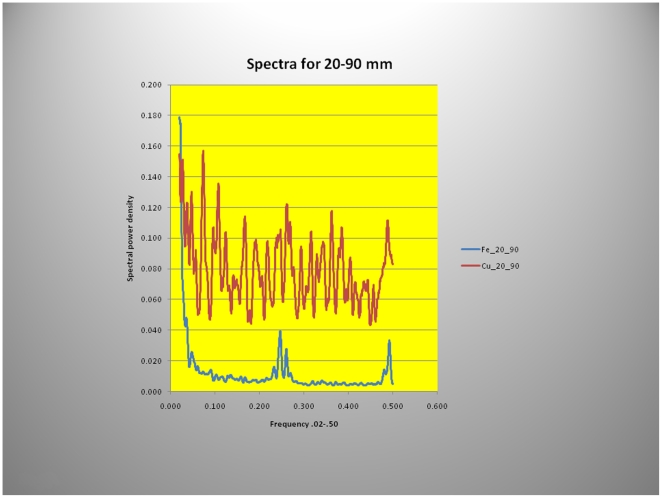
Power spectra of micro-focused synchrotron X-ray fluorescence analyses of Yuk mammoth hair derived from Cu and Fe during the summer months. Notable is the absence of discernable power in the low, mid and high frequencies in the Cu spectrum and the clear spectral peaks derived from Fe in this segment of the hair grown during the Siberian summer (∼4months, 20–90 mm).

**Figure 12:**
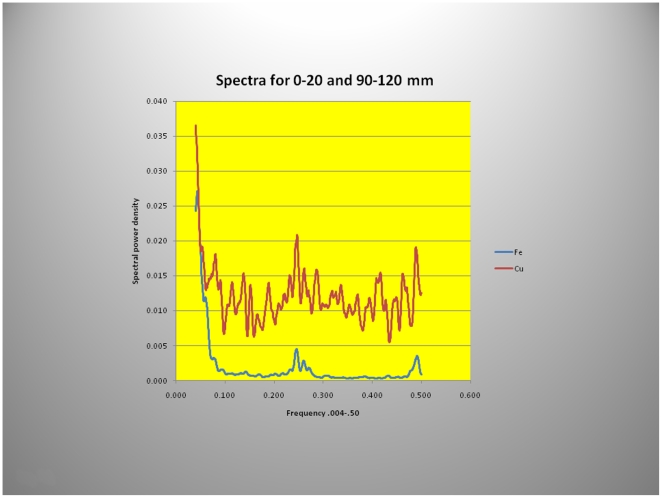
Power spectra of micro-focused synchrotron X-ray fluorescence analyses of Yuk mammoth hair derived from Cu and Fe during the spring and autumn months. The spectra for both seasons have been “stacked” along rows. Discernable power in the low, mid and high frequencies of the spectra obtained from both metals is evident. Cu is a micronutrient and food intake must have increased during these periods to generate sufficient power in the spectra derived from Cu, (see also Fig. 11).

To guide the power spectral analysis of seasonal variations in hair growth (Fig. 10) the two periods of abundant Fe uptake at 5 and 104 mm from the hair root were used, a distance of approximately 9 cm corresponding to ∼4 months of growth (growth-rate, 31 cms/year). Mammoths are thought to have increased their food intake in the spring and fall, before and after the food scarcity in winter or the presumed abundance of food during the summer months. This may account for the two “humps” in the fluorescence intensity (Fig. 10). The power spectra derived from Fe and Cu content depicted in Figs. 11 and 12 shows, for the summer variation in the power derived from Fe for the low-frequency, mid- frequency and high-frequency but only random oscillations for the Cu intake during the same period (Fig. 11). By contrast spring and autumn periods resulted in power spectra derived from the abundance of both metals most likely the result of increased food intake in preparation for the cold months or for migration to warmer areas (Fig. 12).

The chemical content of 3 mammoths' hairs (YUK, JAR and FISH) and of the human hair was determined (Table 1). There were no discernible differences in sulfur content among the mammoth hairs but significant differences in magnesium and calcium were present. The human hair contained much less iron than mammoth hair and more sulfur (Table 1).

**Table 1:** Mean ± SD elemental quantities derived from electron microprobe analysis (k-ratios).

Species	Human	Yuk	Jar	Fish	ANOVA *P value*
S	107.9±9.5^A^	89.3±14.6^AB^	84.3±12.2^B^	79.2±6.7^B^	0.03
Mg	2.2±0.1^C^	7.8±2.0^B^	21.4±3.9^A^	13.6±2.0^D^	<0.001
Ca	7.9±2.4^C^	25.9±0.8^B^	57.9±19.2^A^	23.8±0.3^BC^	<0.001
Fe	0.2±0.4^B^	10.5±5.5^A^	12.1±8.3^A^	12.3±4.3^A^	0.03

P-values by 1-way ANOVA. Means with the same superscript letter (A, B, C, D) are not significantly different in post hoc pair-wise comparisons.

Note that the Fe content among the mammoths' hairs does not differ significantly, but is significantly higher than in human hair.

## Discussion

Until recently the study of biologic rhythms was conceived from the perspective of living organisms. We offer here a glimpse at biologic rhythms as they were in extinct mammoths that lived millennia ago and compare those to results obtained from modern humans.

A major shift in biorhythm research is the availability of new tools such as hydrogen isotope ratios and micro-focused synchrotron X-ray fluorescence analyses which facilitated this look into the past.

Power spectral analysis of heart rate variability (the variation in the duration of heart rate-intervals) is an accepted method to assess the biologic rhythms in living humans [8]. Here, this technique was applied, replacing heart rate intervals with the hydrogen isotope ratios along the length of the hairs.

Hydrogen isotopes, have offered a non-invasive probe into the behavior of living animals. These ratios measured, at intervals along the length of hair, have been found to vary with age of the individuals, with disease and with residence at altitude in contemporaneous humans; they also reflect the geographic “isoscapes” (isotope-landscapes) of the hydrogen isotopes in the food and water consumed [3]. We found that the hydrogen isotope ratios along the length of mammoth's hairs show slow and fast rhythms, as determined by the power spectra of these stable isotope, which varied greatly with the location of the find-spots in Siberia (Figs. 1), supporting the contention that they are a reflection of the hydrogen isotope content of water and the food consumed [9] during life and are proxies for “isoscapes” (isotope-landscapes) or grazing grounds of the mammoths; to some extent they also account for the differences found in the annual fast and slow periods of growth cycles (Fig. 2). The very speedy fast cycling is consistent with the fast mammoth's hair growth; about twice as fast as human hair grows.

To illustrate the relationships between the complex natural influences that control biologic rhythms, such as soil content of nutrients, geography and circadian rhythms (the power spectra derived from hydrogen isotope ratios along the length of the mammoth's hairs) we used Mandelbrot fractal sets (Fig. 3) [9]. Fractals, a term coined by Benoit Mandelbrot, are ubiquitous in nature; they are defined by recurrence relations at each point in a space and are valuable to illustrate complex natural phenomena such as the formation of frost crystals on a glass pane or the contour of mountains and, in biology, the ¾ scaling (M^3/4^) of cellular metabolism, heart beat (here hydrogen isotope ratios along the length of hairs) blood circulation, growth and size, development and life-span [10].

Because in nature, numerous ecological and biological phenomena, such as, river networks and blood vessel branching, are self-similar or fractal-like [11], we generated fractals to illustrate the natural interplay of physical systems, such as soil content of minerals, stable isotope levels in water and food, the power spectra derived from hydrogen isotope ratios in the hair, and the rotation of the earth around the sun, as reflected in the circadian and longer term rhythms during life of the mammoths.

The most Eastern location in Siberia, separated by approximately 4 time zones (∼4600 Km), showed the greatest deviation from the fractal patterns generated from the other two mammoths (Fish and Jar) implying that geography, soil content of nutrients and circadian rhythms have an important determining effect on biologic rhythms (Fig. 3). Additionally, the fractal patterns generated from similar data derived from humans showed marked differences from the mammoths implying that size, which scales with metabolism, is also an important determinant of biologic rhythms (Fig. 3).

We used confocal and scanning electron microscopy to gage the degree of preservation of the tissues. This varied with age and location (Fig. 4.). Notably, mammoths hair preserved in permafrost had less bacterial colonization than contemporaneous human hair (Fig. 4.).

Microfocused synchrotron X-ray fluorescence analyses of the hairs enabled us to identify the concentrated and diffuse distributions of Fe, As and Cu as a function of hair length and thus time (Figs. 5, 6, 7). X-ray fluorescence tomography also showed that mammoth's hairs like that of other modern arctic animals, such as polar bears, have hollow shafts filled with air for better insulation (Fig. 8).

Using X-ray fluorescence data, a clear definition of seasonal variations in food consumption, or migration (Figs. 9, 10) and consequent changes in biologic rhythms were found (Figs. 11, 12). The power spectrum derived from the Cu content during the summer/winter showed no clear discernable power at any frequency (Fig. 11) consistent with the presumed micro-nutrient function of Cu and its reduced food intake and abundance during those periods. By contrast, Fe the most abundant element in the hair (and soil) had clear low, mid- and high frequency power during the same season (Fig. 11). Conversely, during spring and autumn, discernable low, mid- and high frequency power were evident in both spectra (Fig. 12) supporting our contention that the power spectra, derived from the elemental content, also reflect, in part, the quantities of food intake which vary with the seasons and the different seasonal biologic rhythms of the animals.

The elemental content of the hairs (Table 1) were also used as proxy for food intake. Fe showed no differences among the mammoths' but was, not surprisingly, significantly higher in these animals than in the human hair; their food intake must have been several orders of magnitude larger and very different, than that consumed by humans. Sulfur content, a major constituent of hair, is primarily a reflection of hair metabolism and was significantly lower in human hair-a reflection of the smaller size and very different metabolism of humans (Table 1).

Circadian rhythms are driven by earth's rotation and studies have centered on these complex, 24 hour, oscillations. Additional, long-period rhythms, as we have shown here, (Fig. 3), exist that are especially important for large bodied and slower growing animals such as mammoths [12].

In biology there is no observation free model. Every data point rests on some theoretical model of the measurement system. Moreover, the assumptions that a model is based on can be equally important as the accuracy of data [13]. Here power spectral plots were used to model biologic oscillations and find correlations to diet, climate, geographic location and life styles of extinct mega-fauna.

## Materials and Methods

The hair from the Yuribey, Gidan peninsula, Siberian mammoth was found at 71°9^′^ latitude; 76°55^′^ longitude donated by the Smithsonian Institution (R. Purdy) to the University of New Mexico, Department of Earth and Planetary Sciences (Z. D. Sharp) and designated herein “Smith” (no dating available). The hair from the Jarkov Siberian, male mammoth, was found at 73°32^′^ latitude; 105°49^′^ longitude. It was ^14^Carbon dated to ∼20,380 years before present and designated herein “Jar”. Hair from the Yukagir, Siberian, male mammoth, was found at 71°52^′^ latitude; 140°34^′^ longitude. It was ^14^C dated to ∼18,500 years before present and designated herein “YUK”. Hair from the “Fishhook mammoth” was found at 74°08′ latitude; 99°35′ longitude. It was ^14^C dated to ∼20,620 years BP and designated herein “Fish”. The Jarkov, Yukagir and Fishhook mammoths's hairs were donated by The Mammoth Site, Hot Springs, SD. The human hair was donated by an elderly contemporaneous US male for the SEM and con-focal microscopic imaging and a young Italian female, aged 31, for the power spectral analyses, both designated herein “Human”. Written informed consent was obtained from both individuals. The Ethics committee of the NMHEMC Research Foundation reviewed and approved the study.

Microfocused synchrotron X-ray fluorescence analyses were performed at beamline X26A at the National Synchrotron Light Source, Brookhaven National Laboratory, Upton, NY, USA.

Samples were prepared for confocal microscopy by cutting the hair into short segments and mounting them in Prolong Gold mounting medium (Invitrogen) on a microscope slide under a number 1.5 cover-slip. Images show only intrinsic fluorescence of the samples.

Hair samples were coated with gold to provide conductivity for analysis on a JEOL 8200 electron microprobe.

Hydrogen isotope ratios were determined using the continuous-flow-high-temperature-reduction technique.

Computations of growth rate of the hairs, periodicities of the observed oscillations in hydrogen isotope ratios, high and low periodicities from spectra and growth rates were carried out using the SAS programming.

Data derived from each hair were entered into the Mandelbrot set-online generator by Dawid Makiela© (Mandelbrot.ovh.org).

Full methods and associated references are given in Text S1.

## Supporting Information

Text S1 Full methods and associated references.
(DOCX)

## References

[1] Campbell KL, Roberts JEE, Watson LN, Stetefeld J, Sloan AM (2010). Substitutions in woolly mammoth hemoglobin confer biochemical properties adaptive for cold tolerance.. Nature Genetics.

[2] Gibbons A (2010). Tiny time machines revisit ancient life.. Science.

[3] Appenzeller O, Qualls C, Barbic F, Furlan R, Porta A (2007). Stable Isotope Ratios in Hair and Teeth Reflect Biologic Rhythms.. PLoS ONE.

[4] Bernard A, Lécuyer C, Vincent P, Amiot R, Bardet N (2010). Regulation of body temperature by some Mesozoic marine reptiles.. Science.

[5] Marcheva B, Ramsey KM, Buhr ED, Kobayashi Y, Su H (2010). Disruption of the clock components CLOCK and BMAL1 leads to hypoinsulinaemia and diabetes.. Nature.

[6] Akashi M, Soma H, Yamamoto T, Tsugitomi A, Yamashita S (2010). Noninvasive method for assessing the human circadian clock using hair follicles..

[7] Sharp ZD, Atudorei V, Panarello HO, Fernández J, Douthitt C (2006). Hydrogen isotope systematics of hair: archeological and forensic applications.. J Archeolog Sci.

[8] Malliani A, Pagani M, Lombardi F, Cerutti S (1991). Cardiovascular neural regulation explored in the frequency domain.. Circulation.

[9] Mandelbrot BB (1982). The fractal Geometry of Nature.

[10] West BG, Brown JH, Enquist BJ (1997). A general model of the origin of allometric scaling laws in biology.. Science.

[11] Brown JH, Gupta VK, Li Bai-Lian, Milne BT, Restrepo C (2002). The fractal nature of nature: power laws, ecological complexity and biodiversity.. Phil Trans R Soc Lond B.

[12] Bromage TG, Lacruz RS, Hogg R, Goldman HM, McFarlin SC (2009). Lamellar bone is an incremental tissue reconciling enamel rhythms, body size, and organismal life history.. Calcif Tissue Int.

[13] Allen M (2010). Embracing an uncertain future.. Nature.

