# Supplementary material for: Biologic Rhythms Derived from Siberian Mammoths' Hairs
Source: PLoS One. 2011 Jun 29;6(6):e21705. doi: 10.1371/journal.pone.0021705 (PMC3126841; doi:10.1371/journal.pone.0021705)
Supplement: Text S1 — Full methods and associated references. (DOCX) [file pone.0021705.s001.docx]

Supplemental on line material text S1

### Microfocused synchrotron X-ray fluorescence analyses

Microfocused synchrotron X-ray fluorescence analyses were performed at beamline X26A at the National Synchrotron Light Source, Brookhaven National Laboratory, Upton, NY, USA. Beamline X26A utilizes a bending magnet source on the NSLS 2.8 GeV electron storage ring, which operates at a current of 300 mA. For these experiments a monochromatic X-ray beam was used, monochromatized using a Si(111) channel-cut monochromator and tuned to an incident beam energy of 12.5 keV. The beam was focused to a spot size of 5 µm in the vertical x 8 µm in the horizontal using a pair of dynamically-bent, grazing-incidence mirrors, each 100 mm long, arranged in a Kirkpatrick-Baez (KB) geometry. X-ray fluorescence from the mammoth hairs was measured using a combined set of three energy dispersive detectors; a Canberra 9-element HPGe array detector placed 90° to the incident beam within the plane of the storage ring to minimize backgrounds from Compton scattering. The other two detectors are single element Radiant Vortex-EX silicon drift diode detectors also at 90° to the incident beam, but each sitting 45° above and below the plane of the storage ring respectively. The 9-element HPGe array provides an active area of 900 mm^2^ and each silicon drift diode detector has an active area of 50 mm^2^. All eleven detector elements are integrated simultaneously using the XMap series of compact PCI-based digital spectrometers produced by X-ray Instrumentation Associates (XIA) interfaced through EPICS and controlled through in-house client software written in IDL. Incident beam intensity was monitored using an ion-chamber upstream of the focusing optics and all images were corrected for changes in incident beam flux through normalization to the change in ion-chamber counts over time.

Two-dimensional X-ray fluorescence compositional maps were produced of single mammoth hairs by physically sectioning the hair into enough sequential sections to fit on a 35 mm slide mount, each section affixed to a Kapton tape support with enough separation to avoid significant overlap. The compositional maps of entire single hairs utilized continuous stage scanning where full energy dispersive spectra are accumulated to provide an image pixel size of 15 µm with an accumulation time of 50 msec. Areas of the hairs were selected for more detailed imaging with a 4 µm pixel size and a 100 msec accumulation time. At these scan rates and given the solid-angle of collection, detection limits for the elements presented here are on the order of 10 ppm.

Plots of fluorescence intensity versus length along the hair were produced by drawing a region-of-interest polygon around each hair strand and then summing fluorescence counts for each horizontal section as a function of vertical distance. Vertices indicating the hair edges for each region-of-interest were defined programmatically by applying a difference-of-gaussians filter to the Fe fluorescence map and then using the minima to detect the edges. Fluorescence counts were normalized by the calculated diameter of the hair at each vertical position using the width of each region-of-interest at a given row in the map as the diameter of the circle. This is an attempt to compensate for differences in fluorescence due to differences in hair mass that would occur where the hair is physically wider. There are uncertainties, however, in assuming that the hair is spherical and where the hair as mounted on the tape is not perfectly vertical relative to the horizontal scan direction. These effects are relatively small in comparison to the magnitude of oscillation observed in the intensity versus distance plots.

Selected small sections of hair from each mammoth studied were also analyzed by X-ray fluorescence computed microtomography to evaluate the three-dimensional distribution of trace metals in reconstructed cross-sections through the hair. This method eliminates any potential alteration of the sample from sectioning. For these analyses approximately 1 mm long sections were mounted to a Huber goniometer for centering at the focal point of the X-ray beam. Each hair was continuously scanned through the focused beam horizontally to provide a 3 µm pixel size with a 300 msec accumulation time. X-ray transmission through the sample was recorded simultaneously using a p-type, intrinsic, n-type (PIN) photodiode and this signal was used to reconstructed an X-ray absorption tomogram through the sample. Tomographic reconstruction of X-ray fluorescence and attenuation within the sample was performed using a Fast Fourier Transform algorithm implemented in IDL.

### Confocal microscopy

Samples were prepared for confocal microscopy by cutting hair segments and mounting them in Prolong Gold mounting medium (Invitrogen) on a microscope slide under a number 1.5 cover-slip. Images show only intrinsic fluorescence of the samples. The images were acquired on a laser scanning Zeiss LSM510 META confocal system with a 63x/1.4 NA oil immersion objective and a 40x/1.3 NA oil immersion objective. The images were obtained using the Zen 2009 software. Sample fluorescence was excited and collected sequentially in three PMT channels: 405 nm diode laser excitation/420-480 nm emission; 488 nm argon laser excitation/505-530 nm emission and 543 nm helium neon laser excitation/561-657 nm. The resulting channel images are displayed as a merged image.

## Scanning electron microscopy (SEM)

Hair samples were coated with gold to provide conductivity for analysis on a JEOL 8200 electron microprobe at the University of New Mexico, Institute of Meteoritics. Samples were examined at 12 kV accelerating voltage, and X-ray analysis was conducted using wavelength dispersive spectrometers equipped with layered synthetic crystals optimized for light elements. The measured X-ray intensity for each of the elements C, N, O, Na, Mg, Al, Si, S, Ca, Fe, and Se in the sample was divided by the X-ray intensity measured on standards of known composition. The ratio of sample intensity to standard intensity is designated the k-ratio. The k-ratios were then normalized to the k-ratio for sulfur for comparison between different hair samples.

## Hydrogen isotope ratios

##

Hydrogen isotope ratios were determined using the continuous-flow-high-temperature-reduction

Technique [1]. Briefly, hair was wrapped in silver foil and placed into the

combustion chamber of a mass spectrometer using a Carlo Erba AS 200-LS auto-sampler. Three

mm long sections of hair were sampled beginning at the proximal end. The stable isotopic compositions of low mass elements such as hydrogen are reported as “delta” (Ð) values in parts per thousand (‰). Ð values are calculated: in (‰)=(R sample/R standard-1)1000 where “R” is the ratio of the heavy to the light isotope in the sample The stable isotope standard for hydrogen is reported relative to the **S**tandard **M**ean **O**cean **W**ater (SMOW). Isotope composition is reported in relation to this standard which has been defined as 0‰. [2].

## The growth rate of hairs

Where an annual cycle in a time series was evident, this sinusoidal cycle was fit using nonlinear regression and removed from the series. The residual series contains fast and slow periodicities (weeks/cycle) (Fig. 3) computed from the power density. The annual growth rates of hair were computed from the annual sinusoidal cycles

## Periodicities of the observed oscillations

Power spectra of the hydrogen isotope ratios in the hair were computed using an accepted

method [3]. Spectral indices of variability in the hydrogen isotope ratios along the length of the

hair represents the dynamic interaction between the autonomic nervous system’s neural control

of the oscillations and its control of the biologic rhythms.

The autonomic sympathetic modulation are revealed in the low frequencies and the high

frequencies represent the autonomic parasympathetic control of the hydrogen isotope variability

along the length of the hairs.

The exploitation of the mean growing rates of hair and the sampling frequency of our series allows us to estimate the periodicity of the observed oscillations.

## Computing high and low periodicities from spectra and growth rates.

In the time series of hydrogen isotope ratio measurements at multiples of 0.3 cm along a hair taken from a Mammoth there is a partial annual sinusoid evident. The periodicity of this sinusoid is 52 weeks, but to illustrate our computations we use the growth rate of the hair to compute this periodicity. Using the half span of the annual sinusoid, the best fit growth rate for the hair of the function of length along the hair in cm: Mammoth is approximately 32 cm/year. Fitting the annual sinusoid as well as a trend yields the

Predicted dD = -158 -.727*cm +8.69*sin (-.196*cm +3.98),

The frequency of the sinusoid is 0.196 radians/cm. To convert from radians to cycles, we calculate (0.196 radians/cm)/ ($2\pi$radians/cycle) = 0.0312 cycles/cm. Applying the growth rate, our frequency is (0.0312 cycles/cm) x (32 cm/52 weeks) = 0.0192 cycles/week and the reciprocal of frequency gives the corresponding periodicity of 52 weeks/cycle.

We now compute the periodicity of the high frequency spectral peak in the spectrum of the Mammoth hair. The Mammoth hair time series was detrended and had the annual sinusoid removed simultaneously by nonlinear regression. The residual time series retains the gaps that the original series had in terms of not having measurements at every multiple of 0.3 cm, but the three different segments of data can be concatenated in the residual series to form one pooled time series whose spectrum may reveal periodicities lower than the annual one. This spectrum showed a high frequency peak at 0.42 cycles/observation on the x-axis (frequency/$2\pi$axis). The measurement interval is:

dt = 0.3 cm/observation; so we calculate a frequency of (0.42 cycles/observation) /dt =

1.4 cycles/cm. Applying the growth rate, we calculate a frequency of

(1.4 cycles/cm) x (32 cm/ 52 weeks) = 0.862 cycles/week and the reciprocal gives a high frequency periodicity of 1.2 weeks/cycle.

The mammoth hair spectrum also showed another peak at 0.15 cycles/observation. The corresponding periodicity is

$$Period=\frac{dt}{\left(freq\right)\left(growth\right)}=\frac{0.3 cm/obs}{\left(0.15 cycles/obs\right)\left(32 cm/52 wk\right)}=3.25 wk/cycle.$$

For mammoth hair, Nyquist folding frequency = 0.5/0.3 = 1.67 cycle/ cm. This frequency times the growth rate gives a frequency of 1.03 cycles/week with a corresponding periodicity of approximately 1.0 week. Since we do not anticipate important periodicities this low or lower, there should be no fold-back contamination in the above results.

### Human hair

In the time series of hydrogen isotope ratio measurements (dD) every 1.0 cm along a hair there is a clear annual sinusoid over more than two years of data. Fitting the annual sinusoid by nonlinear regression yielded the function of length along the hair in cm:

Predicted dD = -70.17 – 0.096*cm + 1.641*sin(.371*cm + .308).

The frequency of the annual sinusoid is 0.371 radians/cm. The corresponding human hair growth rate= (2π radians/cycle)/ (0.371 radians/cm)/=16.9 cm/year.($2\pi$radians/cycle) = 0.059 cycles/cm. Applying the best fit hair growth rate of 16.9cm/year.

We now compute the periodicity of the high frequency spectral peak in the spectrum. The human hair time series was detrended and had the annual sinusoid removed as described above. This spectrum does showed a high frequency peak at 0.346 cycles/observation on the x-axis (frequency/$2\pi$axis). The measurement interval is dt = 1.0 cm/observation; so we calculate a frequency of (0.346 cycles/observation) /dt =0.346 cycles/cm. Applying the growth rate, we calculate a frequency of (.346 cycles/cm) x (16.9 cm/ 52 weeks) = 0.112 cycles/week and the reciprocal gives a high frequency periodicity of 8.9 weeks/cycle.

The human hair spectrum also showed another peak at 0.192 cycles/observation. The corresponding periodicity is

$$Period=\frac{dt}{\left(freq\right)\left(growth\right)}=\frac{1.0 cm/obs}{\left(0.192 cycles/obs\right)\left(16.9 cm/52 wk\right)}=16 wk/cycle.$$

Another peak frequency of .096 cycles/observation yields a corresponding periodicity of

$$Period=\frac{dt}{\left(freq\right)\left(growth\right)}=\frac{1.0 cm/obs}{\left(0.096 cycles/obs\right)\left(16.9 cm/52 wk\right)}=32 wk/cycle.$$

For human hair, Nyquist folding frequency = 0.5/1.0 = .5 cycle/ cm. This frequency times the growth rate gives a frequency of .163 cycles/week with a corresponding periodicity of 6.2 weeks. The aliases of a given frequency (*f*) are

$f\pm2k Nyquist,for k=\pm1, \pm2, \pm3, \cdots$.

For the high frequency spectral peak at 0.081 cycles/week, the relevant aliases are 0.112 ± 2x.163 cycles/week or -0.213 and 0.437, whose corresponding periodicities are 4.7 and 2.3 weeks. Our computed high frequency peak of periodicity 8.9 weeks is unlikely to be contaminated by our sampling rate. The important aliases for the 0.062 cycles/week peak are -.263 and 0.387, whose corresponding periodicities are 3.8 and 2.6 weeks. Again our 16 week periodicity is unlike to be contaminated. The important aliases for the 0.031 cycles/week peak are -.294 and 0.356, whose corresponding periodicities are 3.4 and 2.8 weeks. Again our 32 week periodicity is unlike to be contaminated.

## Mandelbrot sets

Data derived from each hair were entered into the Mandelbrot set-online generator by Dawid Makiela^©^ (Mandelbrot.ovh.org)

X_1_: Sulfur content; Y_1_: Low frequency/high frequency ratio of power spectrum; X_2_: elemental content (Ca, Mg or Fe); Y_2_: total power of low, mid- and high frequencies; X: total low power; Y: total high power; R: sum of low, mid and high frequencies of the power spectrum obtained from the hydrogen isotope ratios along the length of each hair.

The Functions were Z_n+1_=Z_n_^2^+Z_0_. Maximum number of iterations was 100.

# References

1. Sharp Z D, Atudorei V, Panarello H O, Fernández J, Douthitt C (2006)

Hydrogen isotope systematics of hair: archeological and forensic applications

J Archeolog Sci 30:1709-1716.

2. Anonymous. (2006) USGS Menlo Park California Resources on Isotopes. Fundamentals of Stable Isotope Geochemistry.

3. Malliani A, Pagani M, Lombardi F, Cerutti (1991) Cardiovascular neural regulation explored in the frequency domain. Circulation 84:482-492.
